# Comparison of traditional culture and molecular qPCR for detection of simultaneous carriage of multiple pneumococcal serotypes in African children

**DOI:** 10.1038/s41598-017-04915-y

**Published:** 2017-07-05

**Authors:** Courtney P. Olwagen, Peter V. Adrian, Shabir A. Madhi

**Affiliations:** 1 Department of Science and Technology/National Research Foundation: Vaccine Preventable Diseases, University of the Witwatersrand, Faculty of Health Sciences, Johannesburg, South Africa; 2 Medical Research Council: Respiratory and Meningeal Pathogens Research Unit, University of the Witwatersrand, Faculty of Health Sciences, Johannesburg, South Africa; 3 National Institute for Communicable Diseases: a division of National Health Laboratory Service, Johannesburg, South Africa

## Abstract

*S*. *pneumoniae* is a common colonizer of the human nasopharynx in high income and low-middle income countries. Due to limitations of standard culture methods, the prevalence of concurrent colonization with multiple serotypes is unclear. We evaluated the use of multiplex quantitative PCR (qPCR) to detect multiple pneumococcal serotypes/group colonization in archived nasopharyngeal swabs of pneumococcal conjugate vaccine naive children who had previously been investigated by traditional culture methods. Overall the detection of pneumococcal colonization was higher by qPCR (82%) compared to standard culture (71%; *p *< 0.001), with a high concordance (*kappa* = 0.73) of serotypes/groups identified by culture also being identified by qPCR. Also, qPCR was more sensitive in detecting multiple serotype/groups among colonized cases (28.7%) compared to culture (4.5%; *p* < 0.001). Of the additional serotypes detected only by qPCR, the majority were of lower density (<10^4^ CFU/ml) than the dominant colonizing serotype, with serotype/group 6A/B, 19B/F and 23F being the highest density colonizers, followed by serotype 5 and serogroup 9A/L/N/V being the most common second and third colonizers respectively. The ability of qPCR to detect multiple pneumococcal serotypes at a low carriage density might provide better insight into underlying mechanism for changes in serotype colonization in PCV vaccinated children.

## Introduction

*Streptococcus pneumoniae* is a common colonizer of the human nasopharynx in both high-income and low-middle-income countries, with carriage being a pre-requisite to pathogenesis of pneumococcal disease as well as transmission between hosts^1–5^. To date almost 100 serotypes have been identified through their capsular polysaccharide and there is a paucity of data on concurrent carriage of multiple pneumococcal serotypes due to limitations of traditional recommended culture methods^6^.

Pneumococcal colonization and serotype characterization is normally investigated by culture of the dominant organism followed by serotyping with specific antisera^7^. Despite the ubiquitous use of culture based methods for nasopharyngeal colonization studies, detection of concurrent colonization by other serotypes of a lower density are often missed^8^. Furthermore, traditional culture methods are not quantitative for the colonizing serotypes. Molecular detection of pneumococci in the nasopharynx have several potential advantages over culture based methods, including detection of multiple serotypes from a single sample with high sensitivity, as well as quantitative PCR methods being able to measure the density of colonization and relative proportion of colonizing serotypes^9^. Interrogating beyond identification of only the dominant serotype colonizing children, could also help inform the extent to which the increase in nasopharyngeal non-vaccine serotype colonization following pneumococcal conjugate vaccine (PCV) immunization is driven by unmasking of previously minor colonizing serotypes or replacement acquisition by new serotypes.

In this study we aimed to evaluate the use of multiplex quantitative PCR (qPCR) for the detection and density characterisation of multiple pneumococcal serotypes on nasopharyngeal swabs, from PCV naive black-African children previously investigated by traditional culture methods.

## Results

**Figure 1:**
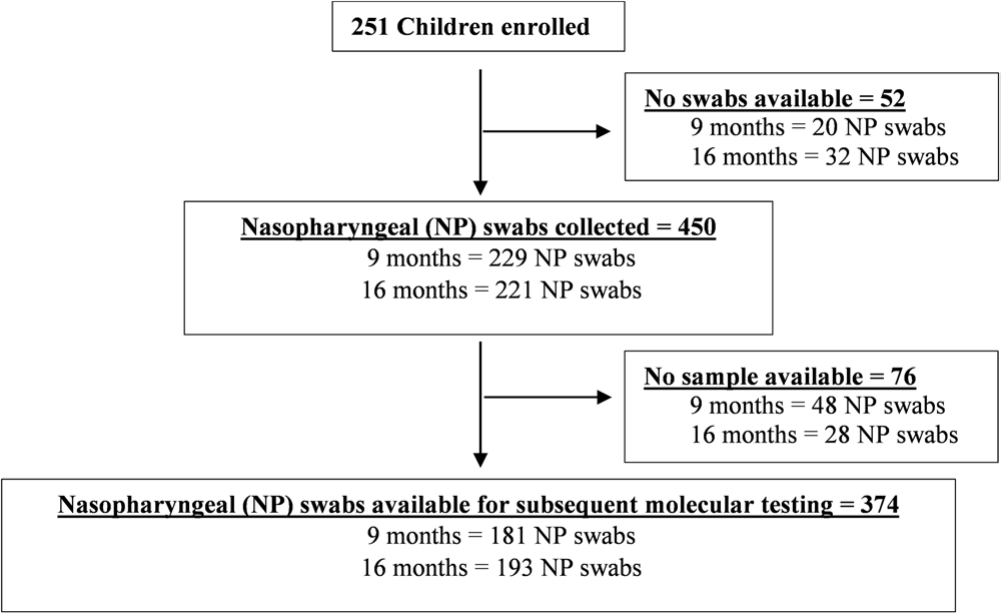
Flow Diagram of study population and sample availability. Diagram indicating the number of children initially enrolled in a PCV-unvaccinated cohort of HIV-uninfected children, as well as the number of nasopharyngeal (NP) swabs available for subsequent molecular qPCR analysis. No swabs were collected due to a missed study visit and the number of NP swabs available for molecular testing were defined by whether there was an adequate volume of sample remaining.

Molecular qPCR analysis involved 374 (83%) of the initial 450 nasopharyngeal swabs collected at either 9 or 15–16 months of age (Fig. 1). All children, in whom samples were available, were black-African, and 49.7% were male. Due to high similarities in findings at both 9 and 15–16 months of age, data was combined for the main analysis.

### Performance of the real-time qPCR assay

All assays were effective in amplifying their respective targets with a high sensitivity, specificity and linearity with the exception of assay 10A/B in which only 10A was detected by the assay. However, the efficiency of all assays ranged from 90% to 105% (Supplementary Table S1). Within the linear dynamic range, the correlation coefficients (r^2^) of all the assays were 0.99. Furthermore, all assays showed high analytical sensitivities with their respective primer and probe pairs with the limit of detection equivalent 10 copies per PCR for all respective assays, with exception to primers/probes that detected serotypes/serogroups 5, 23F, 14, 6A/B/C/D, 13 and 10A in which the LLD was 10 fold less sensitive (100 copies per PCR). All primer pairs and probes were tested with genomic DNA from all pneumococcal and bacterial controls, and no cross reactions occurred (Supplementary Table S2). Both the inter-assay variation (repeatability) and inter-assay variation (reproducibility) for all respective assays was <0.1, while the accuracy for all respective assays was within ± 0.1 (Supplementary Table S1).

### Detection of pneumococcal carriage and serotype detection by culture and qPCR

**Figure 2:**
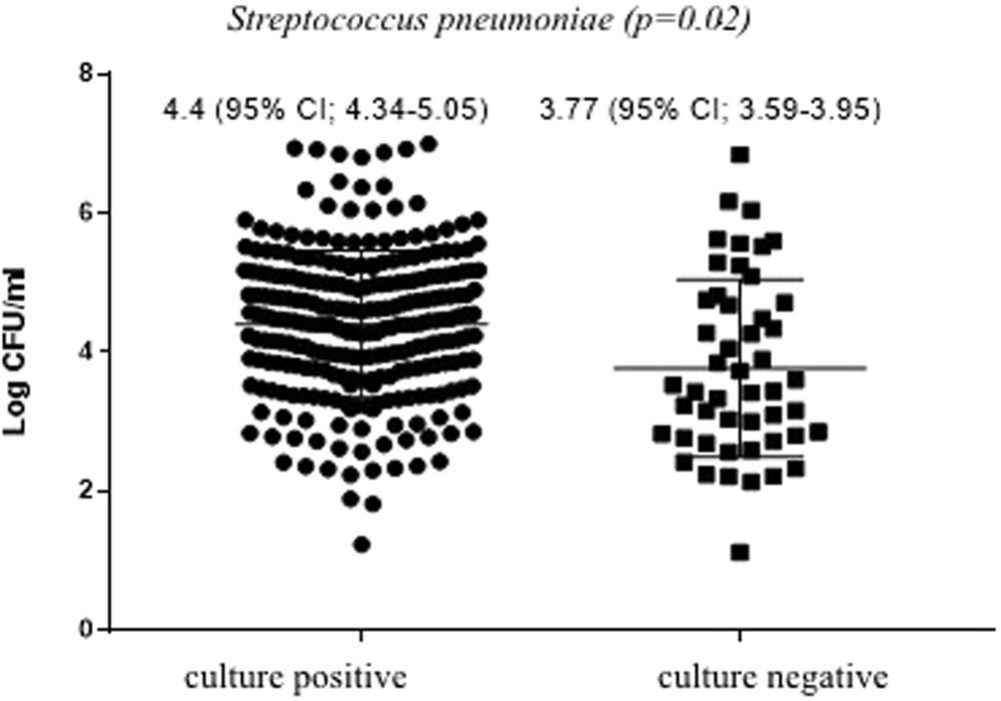
Density of pneumococcal carriage as determined by quantitative PCR (qPCR) grouped by culture positive and culture negative samples.

There was modest concordance between qPCR and culture for the detection of overall pneumococcus from infant NP swabs (*kappa* = 0.58), with the *LytA* qPCR assay being more sensitive than culture (82% vs. 71%; *p* < 0.001); Table 1. The qPCR demonstrated high sensitivity (84%) and specificity (88%) compared to culture used as a referent standard for detection of pneumococcus, with the overall density of pneumococcal carriage being higher in culture-positive (4.4, 95% CI: 4.34–5.05 CFU/ml) than culture-negative samples (3.77; 95% CI: 3.59–3.95 CFU/ml); Fig. 2.

**Table 1:** Yield of culture and molecular qPCR for the detection *Streptococcus pneumoniae* in nasopharyngeal swabs (n = 374).

	qPCR (−)	qPCR (+)	Total	*p*-value	Concordance (*kappa*)	PPV	NPV	Sensitivity (%)	Specificity (%)
Culture (+)	8(2)	258(69)	266(71)	<0.001	0.58	0.97	0.55	84	88
Culture (−)	59(16)	49(13)	108(29)						
Total	67(18)	307(82)	374(100)						

Numbers are value (%), *p*-values of <0.05 were considered significant. PPV, positive predictive value. NPV, negative predictive value.

Discordant results for identification of overall pneumococcus between qPCR and culture were strongly associated with density of pneumococcal carriage (Supplementary Table S3). The majority (47/49; 96%) of qPCR positive but culture-negative samples had estimated copy numbers of <10^4^ colony forming units (CFU)/ml per swab. Similarly, the majority (7/8; 87.5%) of qPCR negative but culture-positive samples were reported as “scant” (<5 colonies/plate) on culture. As pneumococcal density measured by qPCR increased, so did the detection by culture increase, with a perfect concordance (21/21; *kappa* = *1*.0) between culture and qPCR at a carriage density of >10^6^ CFU/ml.

**Figure 3:**
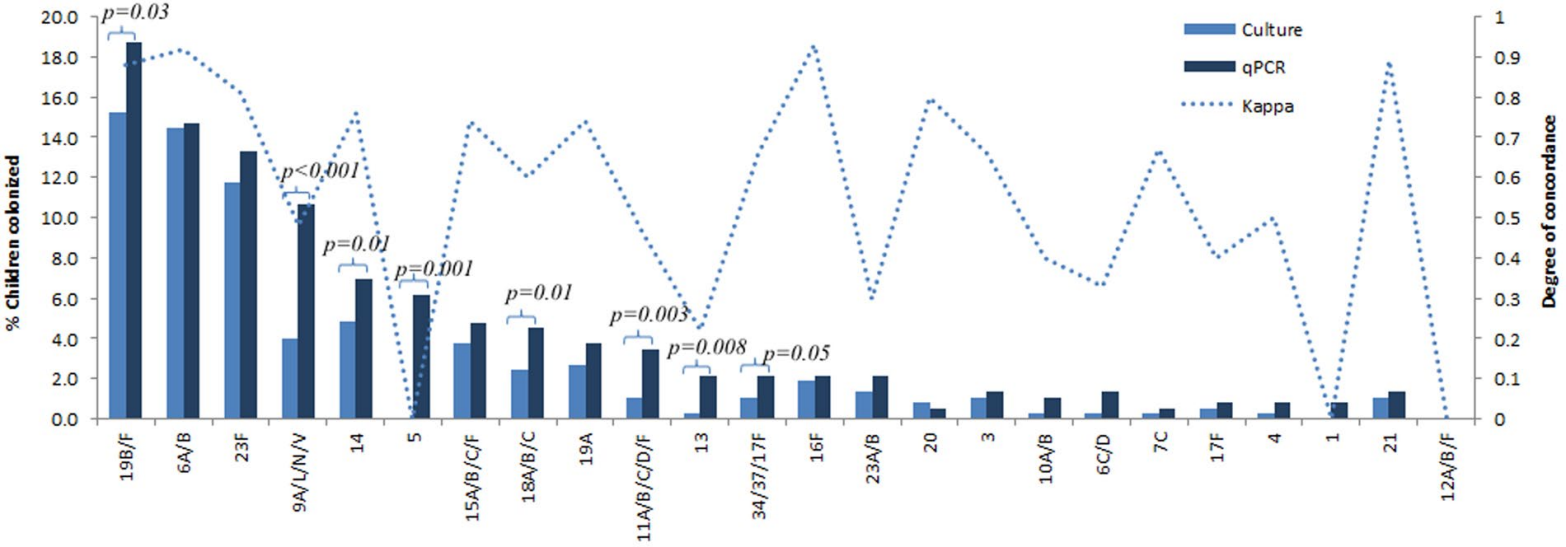
Overall prevalence of nasopharyngeal (NP) pneumococcal colonization as detected by traditional culture and quantitative PCR (qPCR) in a PCV-unvaccinated, HIV-uninfected cohort. Concordance between traditional culture and quantitative PCR (qPCR) were calculated using McNemar’s test with serogroups detected by qPCR included as serotypes. *p*-values of <0.05 were considered significant. Serogroup 6A/B (54): 35.2% Serotype 6A and 64.8% Serotype 6B on culture method. Serogroup 6C/D (1): 100% Serotype 6C on culture method. Serogroup 11/A/B/C/D/F (4): 100% were serotype 11A on culture method. Serogroup 15/A/B/C/F (14): 21%, 64.3%, and 14.2% were serotypes 15A, 15B and 15C on culture method respectively. Serogroup 18A/B/C (9): 100% were serotype 18C on culture method. Serogroup 19B/F (58): 8.6% were serotype 19B and 91.4% were serotype 19F on culture method. Serogroup 23A/B (5): 60% were serotype 23A and 40% were serotype 23B on culture method. Serogroups 34/37/17A (4): 100% were serotype 34 on culture method.

There was a high concordance (*kappa* = 0.73) between serotypes/groups identified by culture and those identified by qPCR, including for those specific serotypes which were individually identifiable on qPCR (*kappa* = 0.68); Supplementary Table S4. The qPCR method, however, identified an additional 150 (57.5%) serotypes/serogroups above those identified by the culture method, including higher detection prevalence by qPCR of serotypes/serogroups 5 (6.61% vs. 0%; *p* < 0.001), 9A/L/N/V (10.7% vs. 4%; *p* < 0.001), 11A/B/C/D/F (3.5% vs. 1.1%, *p* = 0.002), 13 (2.1% vs. 0.3%, *p* = 0.008), 14 (7% vs. 4.8%, *p* = 0.002), 18A/B/C (4.5% vs. 2.4%, *p* = 0.011), 19B/F (18.7% vs. 15.5%, *p* = 0.033) and 34/37/17A (2.1% vs. 1.1%, *p* = 0.05) (Fig. 3), with the sensitivity and specificity for the serotyping qPCR assays shown in Supplementary Table S5. Of these additional serotypes detected by qPCR, 98.8% had densities <10^4^ CFU/ml (Supplementary Table S6). Furthermore, 78% (117/150) of the additional serotypes detected by qPCR were co-carried with other pneumococcal serotypes, with only 12.8% (15/117) of these being the highest density colonizing serotype (Supplementary Table S7).

### Pneumococcal co-colonization

Molecular qPCR was more sensitive than culture in detecting pneumococcal serotypes that were co-carried with two and three or more serotypes detected in 19.2% vs. 4.5% (*p* < 0.001) and 9.4% vs. 0% (*p* < 0.001) of swabs respectively (Table 2). A further 14% (43/307) of *Lyt*A positive NP swabs were negative for all the serogroups/serotypes we tested for, implying they were either non-typable, belonged to one of the serotypes we did not test for, or were *Lyt*A positive non-pneumococcal streptococcal species. However, due to the strong concordance between serotypes identified by qPCR also being identified by culture, we impute that at least 11/43 (25.6%) of NT serotypes identified by qPCR belong to one of the serotypes we did not test for.

**Table 2:** Pneumococcal co-colonization as measured by traditional culture and quantitative molecular PCR (n = 374).

	Culture	qPCR	*p*-value^a^
Not colonized	108(29)	67(18)	<0.001
**Colonized**	266(71)	307(82)	
1 serotype	239(99.8)	176(57.3)	<0.001
2 serotypes	12(4.5)	59(19.2)	<0.001
3+ serotypes	0	29(9.4)	<0.001

Values are number (%). For multiple carriage percentage values are calculated based on total number of samples positive for *LytA*. PCV7, 7-valent pneumococcal conjugate vaccine. ^a^As defined by McNemer’s test. *p*-values of < 0.05 were considered significant.

**Figure 4:**
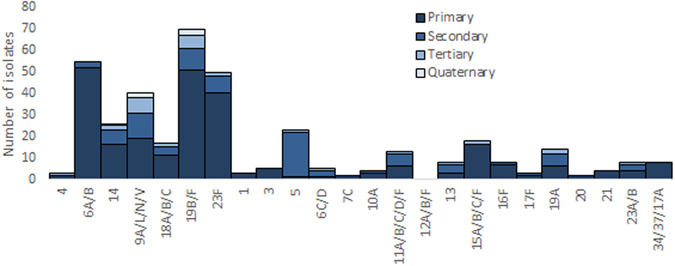
Serotype/group specific ranking of multiple pneumococcal carriage. Each isolate of *S. pneumoniae* was ranked according to its carriage density as determined by quantitative PCR (qPCR) to other isolates present in the same sample. Single colonizers were included in the analysis as primary colonizing serotypes.

**Figure 5:**
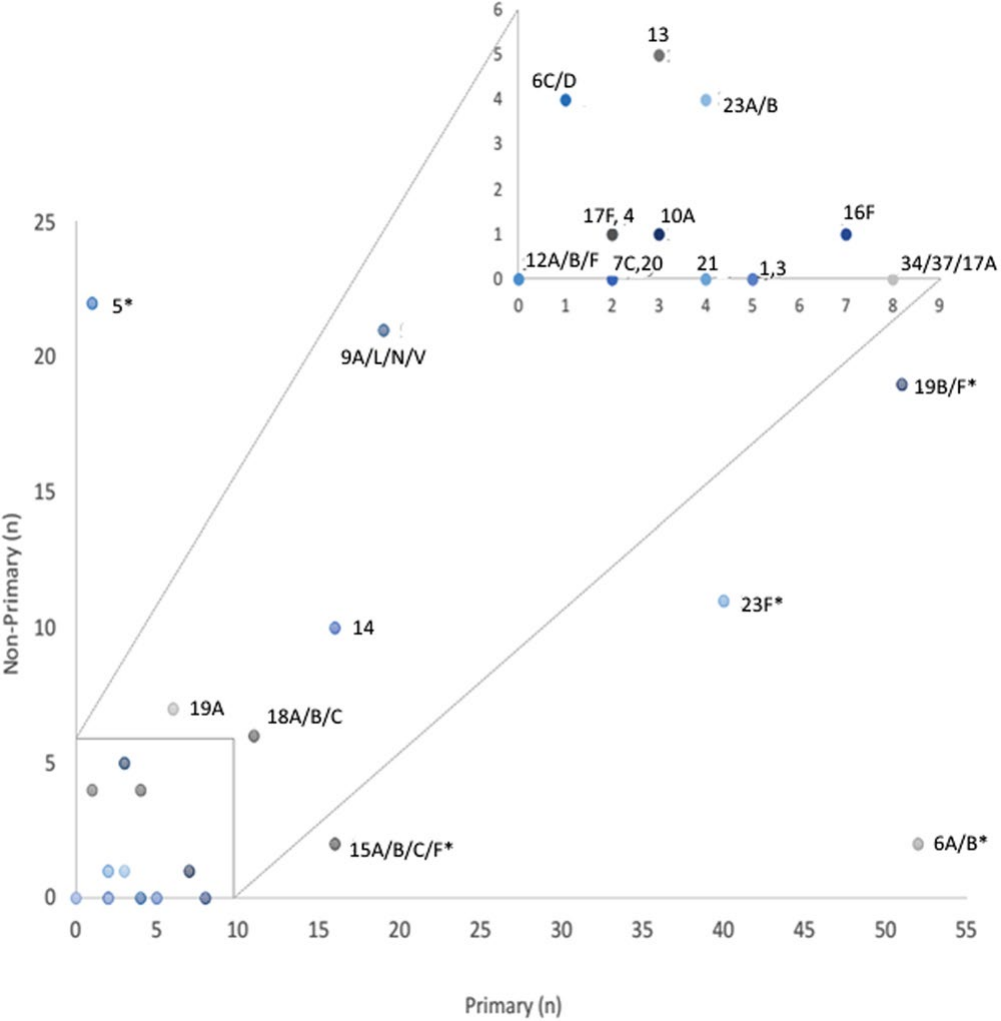
Serotype specific propensity of whether a given serotype/serogroup is more likely to be found as a primary or non-primary isolate. Pneumococcal serotypes identified from nasopharyngeal swabs of black South-African children were classified according to whether they occurred as a primary or non-primary isolate as determined by carriage density. Single colonizers were included in the analysis as primary isolates. The box at the top of the graph is an inset of the blocked region at the bottom. *Results were considered significant when the *p*-value was < 0.05.

Serotypes/serogroups 6A/B, 19B/F, 23F, 5, and 9A/L/N/V were the most common serotypes to be simultaneously co-carried as detected by molecular qPCR (Fig. 4), with serogroups 6A/B and 19B/F being the most common co-colonizers (prevalence 11/88, 12.5%), followed by serogroup 19B/F and serotype 5 (prevalence 8/88, 9.1%). Serotype/groups 6A/B (prevalence 52/264, 19.7%), 19B/F (prevalence 51/264, 19.3%) and 23F (prevalence 40/264, 15.2%) were the highest density colonizers (dominant first colonizing serotype), with serotype 5 (prevalence 21/88, 23.9%) and serogroup 9A/L/N/V (prevalence 7/29, 24%) being the most common second and third colonizers respectively, as determined by density of carriage. Furthermore, serogroup 6A/B was most frequently found as a primary isolate (42/44; 95.5%) with other co-colonizing serotypes, whilst serotypes/group 1, 3, 20, and 34/37/17A were identified as primary isolates only, and serotype 17F and 10A were respectively identified as second and third colonizers only. The serotype specific propensity of whether a given serotype/serogroup is more likely to be found as a primary or non-primary isolate is shown in Fig. 5, with serotypes/serogroups 6A/B (*p* < 0.001), 19B/F *p* < 0.001), 23F (*p* < 0.001), 15A/B/C/F (*p* < 0.001) and 34/37/17A (*p* = 0.008) being more likely to be identified as a primary than a non-primary isolate and serotype 5 being more likely to be identified as a non-primary isolate (*p* < 0.001).

## Discussion

The findings of our study showed that qPCR was more sensitive in detecting concurrent carriage with multiple pneumococcal serotypes, with at least 28.7% of all *LytA* positive nasopharyngeal samples having multiple serogroups/types identified compared to 4.5% of culture positive cases. Furthermore, we were able to quantify the relative proportion of these serotypes and found the majority of the additional serotypes detected by qPCR to have a lower bacterial load (<10^4^ CFU/ml) than the dominant colonizing serotype, indicating why these less dominant serotypes are likely missed by traditional culture methods that may be suboptimal for detecting low density carriage.

Serotypes/serogroups 6A/B, 19B/F, 23F, 5 and 9A/L/N/V were found as common co-colonizers of the nasopharynx. While serotypes/serogroups 6A/B, 19B/F, 23F and 9A/L/N/V are included among PCV7 targeted serotypes, serotype 5 is a non-PCV7 serotype, hence immunization with PCV7 could provide an opportunity for this serotype to occupy the vacant niche and contribute to replacement disease in our setting, as it has also been shown to have high invasive disease potential in South Africa^10, 11^. Further, a study done in Japan found serotype 3 to increase from 0% (2008–2010) to 1.3% (2011–2013) in children <5 years after PCV7 immunization, despite an overall decrease of IPD by 57%^12^. While in the present study we found serotype 3 to be identified as a primary isolate only. Despite the age and geographical difference between these two populations in the two above mentioned studies, the findings support the hypothesis that serotype 3 increased isolation rates after PCV immunization may be due to replacement acquisition of new serotypes.

Several models have demonstrated competition between pneumococcal serotypes for both acquisition and persistence of colonization^13–15^, with more prevalent and higher colonizing density serotypes being less metabolically demanding and thus better able to grow in a nutrient deficient environment. Our findings were in line with those from Dhoubhadel *et al*., in that concurrent colonizing serotypes had different bacterial loads, with one serotype dominating over the other(s), which may explain the competition among concurrent colonizing serotypes for growth due to limited nutrients and space^16^. Furthermore, few studies have been powered to determine if certain pairs of serotypes occur more frequently together, with most finding no significant combinations above the 5% threshold error^14, 17, 18^. Our study, however, found serogroups 6A/B and 19B/F to be the most common concurrent colonizers, occurring in 12.5% of co-colonised children, followed by serogroup 19B/F and serotype 5, which were found in 9.1% of co-colonised children.

A limited number of studies have investigated for concurrent colonization with pneumococcal serotypes in children, with rates varying from 1.5% to 40% according to geographical distribution and method used^6, 19–21^. Findings from most of these studies, however, were limited in that the majority used non quantitative traditional culture based methods that were insensitive in detecting multiple carriage, and therefore likely underestimated the true prevalence of this phenomenon^6^. Concurrent carriage of multiple pneumococcal serotypes in the present study was found in 28.7% of all colonised children. This finding was comparable to a recent study by Wyllie *et al*., which also used qPCR, where concurrent colonization of multiple pneumococcal serotypes was found in 22% of colonised Nepalese children^22^. Other methods have also been used to investigate concurrent colonization such as conventional PCR^23, 24^, colony blot^25^, RFLP^26^ and microarray^27^, with the latter recently been shown to have the best performance overall for pneumococcal serotyping^28^. In studies done by Valente *et al*. and Kandasamy *et al*., microarray found concurrent colonization in 20% and 22% of colonised Portuguese and Nepalese children respectively^18, 29^. While these results are comparable to our findings, Kamng’ona and colleagues found concurrent colonization in 40% of Malawian children by microarray, a higher rate compared to our findings^21^. Our qPCR method, however, had the added benefit in that we were able to quantify the relative proportion of concurrent colonizing serotypes, it did not rely on an initial culture step which might change the relative proportion of the various strains, and it is less labour intensive and less expensive.

Our study was limited in that a) the qPCR method did not detect all pneumococcal serotypes and thus co-carriage in samples colonized with these non-tested serotypes could have been missed and the rate of concurrent colonization could have been underestimated. b) The qPCR method was not able to discriminate between all serotypes within their respective serogroups. While recent studies have shown it is possible to discriminate some serotypes from their respective serogroups further (i.e. 19B and 19F from serogroups 19B/F and serogroups 9A/V and 9L/N from serogroup 9A/L/N/V)^30^, the high genotypic similarities between the capsular loci of certain serotypes still make it difficult to develop some serotype-specific assays and therefore certain serogroups will remain unresolved to specific serotypes. For example, serotype 6A and 6B differ by a sugar linkage due to a point mutation^31^, while serotypes 9A and 9 V have the same syntenty and differ only in their acetylation patterns^32^. Nonetheless, it is epidemiologically important to be able to discriminate serotypes from their respective serogroups, especially in the case where one serotype is included in the pneumococcal conjugate vaccine and the other is not, and the inability of the qPCR method to differentiate these serotypes will be an epidemiological challenge for vaccine effectiveness studies. Nevertheless, due to high concordance between culture and qPCR for the detection of serotypes in our study, the relative proportion of these serotypes could be estimated as similar to the proportion detected by culture. Further, the qPCR method was still able to identify serotypes/groups in culture-negative samples that would have otherwise have been missed; as well as being able to detect multiple serotypes from a single serotype and measure the density of the colonizing serotypes, therefore still making qPCR a useful alternative to traditional based methods. c) We were also not able to compare non-typables detected by culture and qPCR, as the qPCR method was not optimized to test for all pneumococcal serotypes and thus analysis could be misleading as a proportion of the NT detected by qPCR would rather indicate that we did not have the assay to test for all serotypes, rather than it being true pneumococcal NT. d) The loss of sensitivity by qPCR when comparing results to culture was most probably a result of degradation of DNA as a result of long-term storage and/or repeated freezing and thawing of samples. e) A recent study by the PneuCarriage project group found qPCR to have a large number of false positive results when testing pneumococcal spiked samples^28^. While this is concerning, the majority of false positives were for assays not included in our method. Serotype 5, however, was the only serotype to be more commonly isolated as a non-primary than a primary isolate which raises a question of whether this serotype is unable to be cultured or if the qPCR method was giving off false positives for this assay, and thus further investigation is needed. A Cq cut off <35Cqs was implemented, and both positive and negative controls were used for all assays, which should minimize the detection of false positives by the qPCR method.

In conclusion, molecular detection of pneumococcal serotypes was more sensitive than culture and allowed us to detect multiple serotypes. The ability of qPCR to detect pneumococcus at a low carriage density could allow us to gain a better understanding of the impact of PCV immunization on the changes in nasopharyngeal pneumococcal colonization and potential impact thereof on disease prevalence. Future studies could, however, aim to further discriminate additional serotypes from their respective serogroup by including additional testing such as RFLP and sequencing to differentiate between some serotypes that have high genotypic similarities.

## Material and Methods

### Study population

We retrospectively analysed archived nasopharyngeal swab samples collected from a cohort of PCV-unvaccinated, HIV-uninfected infants from Soweto, South Africa, who had previously been investigated by standard culture methods. Detailed information of the study cohort has been described^33, 34^. Briefly, the cohort included 251 PCV naïve infants and their mothers that were enrolled between January 2007 through to October 2007, including 125 infants born to HIV-infected women but who were HIV-uninfected (HEU); and 126 infants born to HIV-uninfected mothers.

Nasopharyngeal (NP) swabs were taken at several time intervals, including at 9 and 15–16 months of age. Swabs were stored in skim milk-tryptone-glucose-glycerol (STGG) transport media at −70 °C at the Respiratory and Meningeal Pathogen Research Unit (RMPRU) in South Africa, as recommended by WHO^35^. The samples were previously cultured for *S*. *pneumoniae* using standard culture methods and pneumococcal serotyping undertaken using the Quellung method as described^36^.

### Multiplex qPCR methods

Stored nasopharyngeal swab samples were thawed and processed. Briefly, NP swabs were vortexed for 30 seconds to release bacteria into the transport media and total nucleic acids were automatically extracted from STGG with the NucliSens® easyMAG® extraction system (BioMérieux, Marcy l’Etoile, France) according to the manufactures instructions. Similarly, total nucleic acids were also extracted from pneumococcal reference strains (positive control strains) grown in Todd-Hewitt broth supplemented with 5% yeast, with the NucliSens® easyMAG® extraction system according to manufactures instructions. Eluted samples and pneumococcal reference strains were stored at −20 °C.

Control strains for the pneumococcal serotypes 1, 2, 3, 4, 5, 6A, 6B, 6C, 6D, 7C, 9A, 9L, 9N, 9V, 10A, 10B, 11A, 11B, 11C, 11D, 11F, 12A, 12B, 12F, 13, 14, 15A, 15B, 15C, 15F, 16F, 17A. 17F, 18A, 18B, 18C, 19A, 19B, 19F, 20, 21, 23A, 23B, 23F, 34 and 37 were obtained from the National Institute for Communicable Diseases (NICD). DNA from these strains were used to optimise PCR assays and as positive controls.

The sequences for oligonucleotide primers and dye-labelled MGB probes were taken from previously published sequences, or designed with the ABI primer express software package, version 3.0 (Applied Biosystems, ABI, Foster City, USA). All primers and probes were designed based on previously published CpsA (Capsular polysaccharide) genes, in which all sequences were obtained from the Gene Bank database (http://www.ncbi.nlm.nih.gov/genbank/). Primer and Probe sequences are described in Supplementary Table S8. All PCV7 serotypes were included as targets in the assays while non-vaccine serotype targets were chosen based on the most frequently isolated non-vaccine serotypes in circulation in South Africa (based on detection by culture methods) at the time of study design. Due to the high genotypic similarities between the capsule loci of certain serotypes, the qPCR method was unable to discriminate between some serotypes within a particular serogroup (i.e. 6A/B, 6C/D, 9A/L/N/V, 10A/B, 11A/B/C/D/F, 12A/B/F, 15A/B/C/F, 18A/B/C, 19B/F, 23A/B and 34/37/17A), but was able to identify serotypes 1, 3, 4, 5, 7C, 13, 14, 16F, 17F, 19A, 20, 21 and 23F individually.

### Primer optimization, standard curves and quantification of the real-time PCR

The qPCRs was optimized in 25 µL reaction volumes containing 12.5 µL 2x TaqMan gene expression master mix (ABI) and 0.25 mM MGB dye labelled probe (ABI) in a primer concentration matrix. Reactions were amplified with the ABI 7500 Real Time PCR system (Applied Biosystems, ABI, Foster City, USA) as per standard manufactures instructions with standard cycling times. Standard concentration curves and limits of detection (LOD) for each target serotype was determined by amplifying three replicates of a 10-fold serial dilution of purified genomic DNA extracted from reference strains with the linear dynamic range being 10^1^–10^7^ copies per standard curve. Target control DNA was harvested from exponential phase cultures (OD_600_ = 0.1), and the number of CFU/swab was estimated from the quantification cycle (Cq) values relative to the standard curve.

The correlation coefficients (r^2^) of each standard curve was determined and the amplification efficiency (e) of a given primer pair was calculated by $\,e={10}^{\frac{-1}{m}}-1$, where m is the slope of the standard curve. Limits of detection (LOD) or analytical sensitivity was determined by the minimum number of copies of DNA that could be measured repeatedly by the primers in the 10-fold serial dilutions with 95% probability. All primer pairs were tested with genomic DNA from all bacterial controls to determine the analytical specificity. Further, intra-assay variation (repeatability) was determined by running the same samples four times in the same assay run and presented as standard deviation (SD) for the copy number of the samples. A ratio of the difference between the observed copy number and the expected copy number was calculated to determine the accuracy of the assays, while the inter-assay variation (reproducibility) was determined by the SD of the copy number of the same sample tested in different runs.

Serotype targets were duplexed (Supplementary Table S9) and paired reactions were tested to ensure that the Cq values did not shift by >1 Cq value, and that sensitivity and specificity remained the same as for singleplex reactions.

### Real-time PCR multiplex assay

Target DNA was pre-screened for pneumococcus by qPCR for the *LytA* gene as described^37^. All *LytA* positive samples (Cq < 35) were regarded as positive for pneumococci and tested for serotypes. Amplification data was analysed with the Applied Biosystems 7500 software, version 2.3 (Foster City, USA) with a manually defined threshold. Negative samples were defined as those with Cq values <35. Furthermore, all *LytA* negative samples were tested for human GAPDH target to confirm the efficiency of the DNA extraction with all qPCR negative samples being positive for GAPDH.

### Statistical analysis

Statistical analysis was performed with STATA Version 11.0 (Statacorp, Texas, USA). All *Lyt*A positive samples in which no serotype was identified were omitted in the multiple carriage analysis. The sensitivities of the qPCR assay and culture were tested with McNemar’s test and when analysed for concordance, all serogroups were included as serotypes detected by qPCR vs. serotypes identified by culture. In the case of non-typable (NT) pneumococcus, we did not compare findings by culture and qPCR, as the qPCR method was not optimized to test for all pneumococcal serotypes and thus analysis could be misleading as a proportion of the NT detected by qPCR could belong to one of the untested serotypes. For density of carriage, geometric mean densities (GMD) and 95% confidence intervals (95% CI) of pneumococcal mean concentrations were calculated following log_10_ transformation on the data. Furthermore, the serotype specific propensity of whether a given serotype/serogroup was more likely to be found as a primary or non-primary isolate, was analysed using a two-tail Binomial test, in which the observed proportion of primary to non-primary isolates were compared. Results were considered significant when the *p*-value was < 0.05.

### Ethical consent

Ethical consent for the initial enrolment was obtained from the Medical Human Research Ethics Committee (HREC) of the University of Witwatersrand (HREC: 050705). Approval for further testing of samples was obtained from the HREC (M120972) and all methods were performed in accordance with the relevant guidelines and regulations. Written, informed consent had been obtained from the parents/guardians of all the study participants at the time of initial enrolment.

### Data availability

The data that support the findings of this study are available from the corresponding author upon reasonable request.

## Electronic supplementary material

Supplementary data
