# Supplementary material for: Comparison of traditional culture and molecular qPCR for detection of simultaneous carriage of multiple pneumococcal serotypes in African children
Source: Sci Rep. 2017 Jul 5;7:4628. doi: 10.1038/s41598-017-04915-y (PMC5498530; doi:10.1038/s41598-017-04915-y)
Supplement: Supplementary file 1 — Supplementary data [file 41598_2017_4915_MOESM1_ESM.pdf]

Supplementary information

Comparison of traditional culture and molecular qPCR for detection of simultaneous carriage of multiple pneumococcal serotypes in African children

Courtney P. Olwagen[1, 2], Peter V. Adrian[1, 2], and Shabir A. Madhi[1, 2, 3*]

[1] Department of Science and Technology/National Research Foundation: Vaccine Preventable Diseases, University of the Witwatersrand, Faculty of Health Sciences, Johannesburg, South Africa.

[2] Medical Research Council: Respiratory and Meningeal Pathogens Research Unit, University of the Witwatersrand, Faculty of Health Sciences, Johannesburg, South Africa.

[3] National Institute for Communicable Diseases: a division of National Health Laboratory Service, Johannesburg, South Africa.

Supplementary table S1: Performance of the quantitative real-time PCR assay

**Bacterial target**	**Efficiency (%)**	**Correlation coefficient (r^2)**	**Limit of detection (copies/PCR)**	**Intra-assay variation**	**Accuracy**	**Inter-assay variation**
Streptococcus pneumoniae (lytA)	97	0.99	10	0.02	0.043	0.025
Streptococcus pneumoniae serotype 1	105	0.99	10	0.011	0.025	0.032
Streptococcus pneumoniae serotype 3	94	0.99	10	0.025	-0.013	0.025
Streptococcus pneumoniae serotype 4	91	0.99	10	0.074	-0.01	0.015
Streptococcus pneumoniae serotype 5	95	0.99	100	0.018	0.048	0.047
Streptococcus pneumoniae serogroup 6A/B/C/D	97	0.99	100	0.013	-0.26	0.023
Streptococcus pneumoniae serogroup 6C/D	92	0.99	10	0.011	-0.078	0.041
Streptococcus pneumoniae serotype 7C	102	0.99	10	0.008	-0.076	0.041
Streptococcus pneumoniae serogroup 9A/L/N/V	97	0.99	10	0.019	-0.059	0.023
Streptococcus pneumoniae serotype 10A/B	91	0.99	10	0.006	0.001	0.008
Streptococcus pneumoniae serogroup 11A/B/C/D/F	98	0.99	10	0.027	0.023	0.053
Streptococcus pneumoniae serogroup 12A/B/F	94	0.99	10	0.018	0.068	0.009
Streptococcus pneumoniae serotype 13	94	0.99	10	0.003	-0.023	0.003
Streptococcus pneumoniae serotype 14	91	0.99	100	0.078	-0.041	0.056
Streptococcus pneumoniae serogroup 15A/B/C/F	98	0.99	10	0.056	-0.059	0.038
Streptococcus pneumoniae serogroup 16F	92	0.99	10	0.03	-0.013	0.029
Streptococcus pneumoniae serotype 17F	99	0.99	10	0.054	-0.026	0.018
Streptococcus pneumoniae serogroup 18A/B/C	91	0.99	10	0.023	0.021	0.047
Streptococcus pneumoniae serotype 19A	92	0.99	10	0.001	0.054	0.005
Streptococcus pneumoniae serogroup 19B/F	93	0.99	10	0.006	0.001	0.003
Streptococcus pneumoniae serotype 20	102	0.99	10	0.015	0.054	0.05
Streptococcus pneumoniae serotype 21	101	0.99	10	0.098	0.075	0.11
Streptococcus pneumoniae serogroup 23A/B/F	96	0.99	10	0.013	-0.056	0.021
Streptococcus pneumoniae serotype 23F	90	0.99	100	0.002	-0.073	0.038
Streptococcus pneumoniae serogroup 34/37/17A	92	0.99	10	0.002	0.09	0.013

Supplementary table S2: Analytical specificity of the quantitative real-time PCR assay

		Bacterial targets																																												
		Streptococcus pneumoniae serotype 1	*Streptococcus pneumoniae* serotype 3	*Streptococcus pneumoniae* serotype 4	*Streptococcus pneumoniae* serotype 5	*Streptococcus pneumoniae* serotype 6A	*Streptococcus pneumoniae* serotype 6B	*Streptococcus pneumoniae* serotype 6C	*Streptococcus pneumoniae* serotype 6D	*Streptococcus pneumoniae* serotype 7C	*Streptococcus pneumoniae* serotype 9A	*Streptococcus pneumoniae* serotype 9N	*Streptococcus pneumoniae* serotype 9L	*Streptococcus pneumoniae* serotype 9V	*Streptococcus pneumoniae* serotype 10A	*Streptococcus pneumoniae* serotype 10B	*Streptococcus pneumoniae* serotype 11A	*Streptococcus pneumoniae* serotype 11B	*Streptococcus pneumoniae* serotype 11C	*Streptococcus pneumoniae* serotype 11D	*Streptococcus pneumoniae* serotype 11F	*Streptococcus pneumoniae* serotype 12A	*Streptococcus pneumoniae* serotype 12B	*Streptococcus pneumoniae* serotype 12F	*Streptococcus pneumoniae* serotype 13	*Streptococcus pneumoniae* serotype 14	*Streptococcus pneumoniae* serotype 15A	*Streptococcus pneumoniae* serotype 15B	*Streptococcus pneumoniae* serotype 15C	*Streptococcus pneumoniae* serotype 15F	*Streptococcus pneumoniae* serotype 16F	*Streptococcus pneumoniae* serotype 17A	*Streptococcus pneumoniae* serotype 17F	*Streptococcus pneumoniae* serotype 18A	*Streptococcus pneumoniae* serotype 18B	*Streptococcus pneumoniae* serotype 18C	*Streptococcus pneumoniae* serotype 19A	*Streptococcus pneumoniae* serotype 19B	*Streptococcus pneumoniae* serotype 19F	*Streptococcus pneumoniae* serotype 20	*Streptococcus pneumoniae* serotype 21	*Streptococcus pneumoniae* serotype 23A	*Streptococcus pneumoniae* serotype 23B	*Streptococcus pneumoniae* serotype 23F	*Streptococcus pneumoniae* serotype 34	*Streptococcus pneumoniae* serotype 37
Assay	1	x																																												
	3		x																																											
	4			x																																										
	5				x																																									
	6ABCD					x	x	x	x																																					
	6CD							x	x																																					
	7C									x																																				
	9ALNV										x	x	x	x																																
	10AB														x																															
	11ABCDF																x	x	x	x	x																									
	12ABF																					x	x	x																						
	13																								x																					
	14																									x																				
	15ABCF																										x	x	x	x																
	16F																														x															
	17F																																x													
	18ABC																																	x	x	x										
	19A																																				x									
	19BF																																					x	x							
	19F																																						x							
	20																																							x						
	21																																								x					
	23ABF																																									x	x	x		
	23F																																											x		
	34/37/17A																															x													x	x

Supplementary table S3: Detection of overall pneumococcal carriage by culture, stratified by colonization density measured by quantitative PCR (qPCR)

Density (CFU/ml)	Number culture positive/ number in density category by PCR (%)
Undetected	8/67 (12)
0-10^2	1/4 (25)
> 10^2-10^3	22/37 (59)
>10^3-10^4	64/81 (79)
>10^4-10^5	85/97 (88)
>10^5-10^6	65/67 (97)
>10^6 -10^7	18/18 (100)
>10^7-10^8	3/3 (100)
Total	266/374 (71)

CFU/ml, colony forming units per millimetre as measured by qPCR. Values are number culture positive/number in density category by qPCR (%).

Supplementary table S4: Yield of culture and molecular qPCR for the detection of *Streptococcus pneumoniae* serotypes/groups, including for those specific serotypes which were individually identifiable on qPCR in nasopharyngeal swabs (n = 374)

	All serotypes/serogroups					Serotypes individually identifiable by qPCR				
	**qPCR (-)**	**qPCR (+)**	**Total**	***p*-value**	**Concordance (*kappa*)**	**qPCR (-)**	**qPCR (+)**	**Total**	***p*-value**	**Concordance (*kappa*)**
Culture (+)	239	21	260			86	11	97		
Culture (-)	150	8566	8562	<0.001	0.73	69	5070	4985	<0.001	0.68
Total	389	8586	8976			155	5080	5236		

Supplementary table S5: Detection of pneumococcal serotypes by culture and quantitative PCR (qPCR)

	Total (N=374)				
**Serotype**	**Culture**	**qPCR**	***p*-value**	**sensitivity**	**specificity**
1	0	3(0.8)	0.08	0	100
3	4(1.1)	5(1.4)	0.56	60	99.8
4	1(0.3)	3(0.8)	0.16	33.3	100
5	0	23(6.1)	<0.001	0	100
6A/B	54(14.4)	55(14.7)	0.99	93	99
A	19(5.1)	-	-	-	-
B	35(9.4)	-	-	-	-
6C/D	1(0.3)	5(1.3)	0.046	20	100
C	1(0.3)	-	-	-	-
D	0	-	-	-	-
7C	1(0.3)	2(1)	0.32	50	100
9A/L/N/V	15(4)	40(10.7)	<0.001	35	99.7
A	0	-	-	-	-
L	0	-	-	-	-
N	2(0.53)	-	-	-	-
V	14(3.7)	-	-	-	-
10A	1(0.3)	4(1.1)	0.08	25	100
11A/B/C/D/F	4(1.1)	13(3.5)	0.003	31	100
A	4(1.1)	-	-	-	-
B	0	-	-	-	-
C	0	-	-	-	-
D	0	-	-	-	-
F	0	-	-	-	100
12A/B/F	0	0	-	-	-
A	0	-	-	-	-
B	0	-	-	-	-
F	0	-	-	-	-
13	1(0.3)	8(2.1)	0.008	12.5	100
14	18(4.8)	26(7)	0.01	65.4	99.7
15A/B/C/F	14(3.7)	18(4.8)	0.16	66.7	99.4
A	3(0.8)	-	-	-	-
B	9(2.4)	-	-	-	-
C	2(0.5)	-	-	-	-
F	0	-	-	-	-
16F	7(1.9)	8(2.1)	0.32	87.5	100
17F	2(0.53)	3(0.8)	0.56	33.3	99.7
18A/B/C	9(2.4)	17(4.5)	0.01	47.1	99.7
A	1(0.3)	-	-	-	-
B	0	-	-	-	-
C	8(2.1)	-	-	-	-
F	0	-	-	-	-
19A	10(2.7)	14(3.7)	0.1	64.3	99.7
19B/F	57(21.9)	70(18.7)	0.034	81.4	100
B	5(0.8)	-	-	-	-
F	52(20)	-	-	-	-
20	3(0.8)	2(0.53)	0.32	100	99.7
21	5(0.8)	4(1.1)	0.32	100	99.7
23AB	5(0.8)	8(2.1)	0.32	25	99.2
A	3(0.8)	-	-	-	-
B	2(0.53)	-	-	-	-
23F	44(11.8)	50(13.4)	0.13	78	98.5
34/37/17F	4(1.1)	8(2.1)	0.046	50	100
34	4(1.1)	-	-	-	-
37	0	-	-	-	-
17A	0	-	-	-	-

Supplementary table S6: Detection of pneumococcal serotype carriage by culture, stratified by colonization density measured by quantitative PCR (qPCR)

Pneumococcal density by qPCR (CFU/ml)	Number of culture positive/number in density category by qPCR (%)											
**Serotype/group**	**1**	**3**	**4**	**5**	**6A/B**	**6C/D**	**7C**	**9A/L/N/V**	**10A**	**11A/B/C/D/F**	**12A/B/F**	**13**
Undetected	0/371	1/369(0.3)	0/371	0/351	3/319(0.9)	0/369	0/372	1/333(0.3)	0/370	0/361	0/374	0/366
0- 10^2	0	0	0/1	0	0	0	0/1	0/19	0	0/7	0	0
> 10^2-10^3	0/2	0/1	0/1	0/23	7/7(100)	0/2	0	1/8(13)	0	0/2	0	0/5
>10^3-10^4	0	1/1(100)	1/1(100)	0	12/16(100)	0/2	1/1(100)	7/7(100)	0/1	2/2(100)	0	0/1
>10^4-10^5	0/1	1/2(50)	0	0	18/18(100)	0	0	5/6(83)	1/1(100)	2/2(100)	0	0/1
>10^5-10^6	0	1/1(100)	0	0	9/9(100)	0	0	1/1(100)	0/2	0	0	0
>10^6 -10^7	0	0	0	0	5/5(100)	1/1(100)	0	0	0	0	0	1/1(100)
>10^7-10^8	0	0	0	0	0	0	0	0	0	0	0	0
>10^8	0	0	0	0	0	0	0	0	0	0	0	0
Total	0/374	4/374(1.1)	1/374(0.3)	0/374	54/374(14.4)	1/374(0.3)	1/374(0.3)	15/374(0.3)	1/374(0.3)	4/374(1.1)	0/374	1/374(0.3)

**Serotype/group**	**14**	**15A/B/C/F**	**16F**	**17F**	**18A/B/C**	**19A**	**19B/F**	**20**	**21**	**23A/B**	**23F**	**34/37/17A**
Undetected	1/348(0.9)	2/356(0,6)	0/366	1/371(0,3)	1/357(0,3)	1/360(0.3)	0/304	1/372(0.3)	1/370(0.3)	3/365(0.8)	5/324(1.5)	0/366
0- 10^2	0	0	0	0/1	0/1	0/2	10/12(83.3)	0	0	0	0	1/2(50)
> 10^2-10^3	2/11(18)	1/3(33.3)	3/3(100)	0/1	1/7(14.3)	1/3(33.3)	8/12(66.7)	0	2/2(100)	2/8(25)	17/26(65.4)	1/4(25)
>10^3-10^4	10/10(100)	5/9(55.6)	3/3(100)	1/1(100)	4/5(80)	8/9(88.9)	21/23(91.3)	0	0	0/1	15/16(93.8)	1/1(100)
>10^4-10^5	4/4(100)	6/6(100)	0/1	0	3/4(75)	0	15/18(83.3)	2/2(100)	0	0	7/8(87.5)	1/1(100)
>10^5-10^6	1/1(100)	0	0	0	0	0	3/4(75)	0	2/2(100)	0	0	0
>10^6 -10^7	0	0	0	0	0	0	1/1(100)	0	0	0	0	0
>10^7-10^8	0	0	1/1(100)	0	0	0	0	0	0	0	0	0
>108	0	0	0	0	0	0	0	0	0	0	0	0
Total	18/374(4.8)	14/374(3.7)	7/374(1.9)	2/374(0.5)	9/374(2.4)	10/374(2.7)	58/374(15.5)	3/374(0.8)	5/374(1.3)	5/374(1.3)	44/374(13.4)	4/374(1.1)

Supplementary table S7: Yield of additional serotypes detected by quantitative PCR (qPCR) in relation to being co-carriage with other pneumococcal serotypes

Serotype/group	Number of serotypes co-carried/additional serotypes detected by qPCR (%)	Number of dominating colonizing serotype/number of serotypes co-carried (%)*
1	2/3(75)	1/2(50)
3	2/2(100)	2/2(100)
4	2/2(100)	0/2
5	23/23(100)	1/23(4.3)
6A/B	3/4(75)	2/3(66.7)
6C/D	4/4(100)	0/4
7C	0/1	0
9A/L/N/V	19/26(73)	0/19
10A	2/3(75)	1/2(50)
11A/B/C/D/F	7/9(77.8)	2/7(28.6)
12A/B/F	0	0
13	6/7(85.7)	0/6
14	7/9(77.8)	1/7(14.3)
15A/B/C/F	5/6(83.3)	1/5(25)
16F	0/1	0
17F	1/2(50)	0/1
18A/B/C	7/9(77.8)	1/7(14.3)
19A	3/5(60)	0/3
19B/F	12/13(92.3)	2/12(16.7)
20	0	0
21	0	0
23A/B	5/6(83.3)	1/5(20)
23F	6/11(54.5)	0/8
34/37/17A	1/4(25)	0/1
Total	117/150	15/117(12.8)

*Density of pneumococcal carriage was used to determine if the additional serotypes detected only by quantitative PCR (qPCR) were dominant colonizing serotypes (highest density colonizer).

Supplementary table S8: Primers and probe sequences for the qPCR detection of pneumococcal serotypes

Primers/Probe[a] name	Sequence (5′ - 3′)	serotype/serogroup	Source
lytA-Fwd<br>lytA-Rev<br>lytA-Probe	TCTTACGCAATCTAGCAGATGAAGC<br>GTTGTTTGGTTGGTTATTCGTGC<br>(VIC)-TTTGCCGAAAACGCTTGATACAGGG-(MGB)	*Streptococcus pneumoniae*	McAvin *et al*, 2001
1-Fwd<br>1-Rev<br>1-Probe	CGTGCGGTAATTGAAGCTATGA<br>TGTGGCCCCAGCAACTCT<br>(FAM)-TGCTTGCCCTTGTATAGGGT-(MGB)	*Streptococcus pneumoniae*_serotype 1	Azzari *et al*, 2010
3-Fwd<br>3-Rev<br>3-Probe	GGTCAGCAGAAAGTATGCATTGG<br>TCGTTTATCCAGGGTCTGATGA<br>(VIC)-TATTGGATGTGGTTTATCGTGAAGA-(MGB)	*Streptococcus pneumoniae*_serotype 3	Azzari *et al*, 2010
4-Fwd<br>4-Rev<br>4-Probe	TGGGATGACATTTCTACGCACTA<br>CCGTCGCTGATGCTTTATCA<br>(FAM)-TCCTATTGGATGGTTAGTTGGTGA-(MGB)	*Streptococcus pneumoniae*_serotype 4	Azzari *et al*, 2010
5-Fwd<br>5-Rev<br>5-Probe	TTACGGGAGTATCTTATGTCTTTAATGG<br>CAGCATTCCAGTAGCCTAAAACTAGA<br>(VIC)-TTGTCTCAGCAACTCTATTTGGCTGTGGG-(MGB)	*Streptococcus pneumoniae*_serotype 5	Azzari *et al*, 2010
6A/B/C/D-Fwd<br>6A/B/C/D-Rev<br>6A/B/C/D-Probe	AAGTTTGCACTAGAGTATGGGAAGGT<br>ACATTATGTCCRTGTCTTCGATACAAG<br>(VIC)-TGTTCTGCCCTGAGCAACTGG-(MGB)	*Streptococcus pneumoniae*_serogroup 6A/B/C/D	Azzari *et al*, 2010
6C/D-Fwd<br>6C/D-Rev<br>6C/D-Probe	TTGGGATGATTGGTCGTATTAG<br>CTCTTCAATTAGTTCTTCAGTTCG<br>(FAM)-CCACGCAATTCGCCATC-(MGB)	*Streptococcus pneumoniae*_serogroup 6C/D	Azzari *et al*, 2010
7C-Fwd<br>7C-Rev<br>7C-Probe	CGTCAGGAATAGGTGCAATCTCT<br>TGAAATTCCAAGCGAAGCAA<br>(VIC)-TTCATCTATTGGTTCTTATGGTGTT-(MGB)	*Streptococcus pneumoniae*_serotype 7C	This Study
9A/L/N/V-Fwd<br>9A/L/N/V-Rev<br>9A/L/N/V-Probe	TGGAATGGGCAAAGGGTAGTA<br>TCGGTTCCCCAAGATTTTCTC<br>(FAM)-TTAATCATGCTAACGGCTCATCGA-(MGB)	*Streptococcus pneumoniae*_serogroup 9A/L/N/V	Azzari *et al*, 2010
10A/B-Fwd<br>10A/B-Rev	CCTCTCCTATCAACTATTACTCATTATACTACCT<br>AATAACCATAAGTCCCTAGATCATTCAAAG	*Streptococcus pneumoniae*_serotype 10A/B	Azzari *et al*, 2010

10A/B-Probe	(VIC)-TCATTACAACTCCCTATGTGACACGGGTCTTTT-(MGB)		
11A/B/C/D/F-Fwd<br>11A/B/C/D/F-Rev<br>11A/B/C/D/F-Probe	ACCGCATTTCTTATCGCACTATATT<br>TCTCCTTACCATCAAACATGTTAATCA<br>(FAM)-TGAATCAGTCTGACCGTTT-(MGB)	*Streptococcus pneumoniae*_serogroup 11A/B/C/D/F	This Study
12A/B/F-Fwd<br>12A/B/F-Rev<br>12A/B/F-Probe	GATTATTCGCTTGCCTCTTCATG<br>ATAGCCGAAATAAGCTTTCCAGAA<br>(FAM)-ATTTGTAAGCGGACGTGCGATT-(MGB)	*Streptococcus pneumoniae*_serogroup 12A/B/F	Azzari *et al*, 2010*
13-Fwd<br>13-Rev<br>13-Probe	TCGGATTTAGTAGTAACCCCATTGA<br>TTCTTGATTGAGGATGCATTTCC<br>(VIC)-AGTAGTAAGAGATCATATTCAAG-(MGB)	*Streptococcus pneumoniae*_serotype 13	This Study
14-Fwd<br>14-Rev<br>14-Probe	CGACTGAAATGTCACTAGGAGAAGAT<br>AATACAGTCCATCAATTACTGCAATACTC<br>(VIC)-TGTCATTCGTTTGCCAATACTTGATGGTCTC-(MGB)	*Streptococcus pneumoniae*_serotype 14	Azzari *et al*, 2010
15A/B/C/F-Fwd<br>15A/B/C/F-Rev<br>15A/B/C/F-Probe	TTGAATCAGGTAGATTGATTTCTGCTA<br>CTCTAGGAATCAAATACTGAGTCCTAATGA<br>(FAM)-CTCCGGCTTTTGTCTTCTCTGT-(MGB)	*Streptococcus pneumoniae*_serogroup 15A/B/C/F	Azzari *et al*, 2010
16F-Fwd<br>16F-Rev<br>16F-Probe	GCAACTGGTATTTTTGATATTGGAGAA<br>CAAAGGAATGCCATGCCATA<br>(NED)-AAAATGCTAACTTCGTTGGAGG-(MGB)	*Streptococcus pneumoniae*_serotype 16F	This Study
17F-Fwd<br>17F-Rev<br>17F-Probe	GTAAAGATTTCATGTCCTATAAGGGAGAA<br>AGGCGTCCCTGTTTATGAGAAG<br>(FAM)-TTGTACATGGTCTGGATTT-(MGB)	*Streptococcus pneumoniae*_serotype 17F	This Study
18A/B/C-Fwd<br>18A/B/C-Rev<br>18A/B/C-Probe	CCTGTTGTTATTCACGCCTTACG<br>TTGCACTTCTCGAATAGCCTTACTC<br>(FAM)-AACCGTTGGCCCTTGTGGTGGA-(MGB)	*Streptococcus pneumoniae*_serogroup 18A/B/C	Azzari *et al*, 2010
19A-Fwd<br>19A-Rev<br>19A-Probe	TTCGACGACGTATCAGCTTCA<br>TCATTGAGAGCCTTAACCTCTTCA<br>(VIC)-ACCCAAAACGGTTGACGCATTATACT-(MGB)	*Streptococcus pneumoniae*_serotype 19A	Azzari *et al*, 2010
19B/F-Fwd<br>19B/F-Rev<br>19B/F-Probe	GGTCATGCGAGATACGACAGAA<br>TCCTCATCAGTCCCAACCAATT<br>(VIC)-ACCTGAAGGAGTAGCTGCTGGAACGTTG-(MGB)	*Streptococcus pneumoniae*_serogroup 19B/F	Azzari *et al*, 2010

20-Fwd<br>20-Rev<br>20-Probe	AAAGATACTGGCTGAGGAGCTATCTATT<br>AGTCAAAAGTACTCAACCATTCTGATATATTC<br>(VIC)-AGGATAAGGTCTACTTTGTGGGAGTTC-(MGB)	*Streptococcus pneumoniae*_serotype 20	Azzari *et al*, 2010
21-Fwd<br>21-Rev<br>21-Probe	CCATTTGAAGGACCAGTTGTTG<br>AAAAAGCCACTATCAGGAATACCAA<br>(FAM)-AATGGCATTGCTTCGTAAA-(MGB)	*Streptococcus pneumoniae*_serotype 21	This Study
23A/B/F-Fwd<br>23A/B/F-Rev<br>23A/B/F-Probe	GGTGGACTTTCCGATGCAA<br>CACTGTCAACAAAAATGAGGTAATCTC<br>(VIC)-AAATGTCGGTATAGATAAAG-(MGB)	*Streptococcus pneumoniae*_serogroup 23A/B/F	This Study
23F-Fwd<br>23F-Rev<br>23F-Probe	TGCTATTTGCGATCCTGTTCAT<br>AGAGCCTCCGTTGTTTCGTAAA<br>(FAM)-TTTCTCCGGCATCAAACGTTAAG-(MGB)	*Streptococcus pneumoniae*_serotype 23F	Azzari *et al*, 2010
34/37/17A-Fwd<br>34/37/17A-Rev<br>34/37/17A-Probe	GGATACTATGTACGAACAGATGGACTTG<br>CTCACTAACTCGCCCGAATAAAC<br>(FAM)-CCGACTATACTCCATTTGA-(MGB)	*Streptococcus pneumoniae*_serogroup 34/37/17A	This Study

Probe[a]: minor grove binding (MGB) dye labelled TaqMan probe. * probe sequence modified.

Supplementary table S9: Primer/probe duplex combinations

Primmer/probe combinations	Duplex reaction
5 & 21	A
20 & 23F	B
4 & 14	C
3 & 17F	D
1 & 23A/B/F	E
6A/B/C/D & 6C/D	F
10A/B & 15A/B/C/F	G
13 & 9A/L/N/V	H
19A & 18A/B/C	I
19B/F & 12A/B/F	J
11A/B/C/D/F & 16F	K
7C & 34/37/17A	L
